# Identification of the nucleotide substitutions in 62 SARS-CoV-2 sequences from Turkey

**DOI:** 10.3906/biy-2005-69

**Published:** 2020-06-21

**Authors:** Ayşe Banu DEMİR, Domenico BENVENUTO, Hakan ABACIOĞLU, Silvia ANGELETTI, Massimo CICCOZZI

**Affiliations:** 1 Department of Medical Biology, Faculty of Medicine, İzmir University of Economics, İzmir Turkey; 2 Unit of Medical Statistics and Molecular Epidemiology, University Campus Bio-Medico of Rome, Rome Italy; 3 Department of Medical Microbiology, Faculty of Medicine, İzmir University of Economics, İzmir Turkey; 4 Unit of Clinical LaboratoryScience, University CampusBio-Medico of Rome, Rome Italy

**Keywords:** SARS-CoV-2, evolution, mutation, COVID-19

## Abstract

A previously unknown coronavirus, severe acute respiratory syndrome coronavirus 2 (SARS-CoV-2), has been shown to cause coronavirus disease 2019 (COVID-19) pandemic. The first case of COVID-19 in Turkey has been declared in March 11th, 2020 and from there on, more than 150,000 people in the country have been diagnosed with the disease. In this study, 62 viral sequences from Turkey, which have been uploaded to GISAID database, were analyzed by means of their nucleotide substitutions in comparison to the reference SARS-CoV-2 genome from Wuhan. Our results indicate that the viral isolates from Turkey harbor some common mutations with the viral strains from Europe, Oceania, North America and Asia. When the mutations were evaluated, C3037T, C14408T and A23403G were found to be the most common nucleotide substitutions among the viral isolates in Turkey, which are mostly seen as linked mutations and are part of a haplotype observed high in Europe.

## 1. Introduction

Coronaviruses (CoV) are enveloped positive-stranded RNA viruses that belong to the family *Coronaviridae* and are divided into 4 genera which are alpha-CoV, beta-CoV, gamma-CoV, and delta-CoV. Similar to severe acute respiratory syndrome coronavirus (SARS-CoV) and Middle East respiratory syndrome coronavirus (MERS-CoV), SARS-CoV-2 is a beta-coronavirus (Gorbalenya et al., 2020). It encodes several structural proteins including envelope (E), membrane (M), nucleocapsid (N) and spike (S) proteins as well as non structural ones (Gorbalenya et al., 2020). The virus was shown to have gone through certain mutations both in its structural and non structural proteins within several months while spreading throughout the world (Pachetti et al., 2020; Wang et al., 2020).

Starting from December 2019, SARS-CoV-2 led to a worldwide COVID-19 pandemic, which caused more than 3 million cases along with more than 250,000 deaths within 5 months^1^1Worldometers (2020). Global COVID-19 statistics [online]. Website https://www.worldometers.info/coronavirus/ [accessed 05 May 2020].. The first case of COVID-19 in Turkey was announced in March 11th and as of May 24th, the number of positive cases and deaths reached to 156,827 and 4,340, respectively^2^2T.C. Sağlık Bakanlığı (2020). Türkiye’deki Güncel Durum [online]. Website https://covid19.saglik.gov.tr/ [accessed 05 May 2020].. A total of 63 sequences from SARS-CoV-2 isolates of Turkey were uploaded to global initiative on sharing all influenza data (GISAID) database between the dates March 25th and May 22nd^3^3GISAID (2020). Website https://www.gisaid.org/ [accessed 24 April 2020].. 

The aim of this study is to reveal the most common mutations of SARS-CoV-2 viral isolates from Turkey in comparison to the reference sequence from China (NC_045512.1). Our results revealed that some of the viral mutations are present in more than 60% of the isolates. Although further analysis and characterizations are needed, the data in this study may contribute to understanding the molecular evolution of SARS-CoV-2 in Turkey.

## 2. Materials and methods

### 2.1. Dataset construction

All the SARS-CoV-2 whole genome sequences have been downloaded from GISAID database3 (Elbe and Buckland-Merrett, 2017; Shu and McCauley, 2017). The whole genome sequence dataset was constructed as including 63 viral sequences from Turkish patients that were submitted to the database between March 25th and May22nd, 2020 (Supplementary Table 1) and the reference SARS-CoV-2 sequence isolated in Wuhan which was downloaded from GeneBank (NC_045512.1)^4^4NCBI (2020). GeneBank Severe acute respiratory syndrome coronavirus 2 isolate Wuhan-Hu-1, complete genome [online]. Website https://www.ncbi.nlm.nih.gov/nuccore/NC_045512.2 [accessed 24 April 2020].. One of the sequences (EPI_ISL_435057) was excluded from the analysis due to harboring extreme number of unique mutations, which can result from sequencing errors.

**Table 1:** Common nucleotide substitutions in 62 SARS-CoV-2 viral genomes fromTurkey (submitted to GISAID between March 25th and May 22nd 2020) compared to the SARS-CoV-2 NCBI reference genome NC 045512.1. The viral gene and gene products were identified according to the reference sequence information from GeneBank4. The nucleotide sequences were indicated starting from the 5’ UTR, while the corresponding amino acid changes were mentioned separately for each protein coding region specific for the corresponding protein.

Nucleotide substitution at the given position	Corresponding viral gene	Corresponding viral gene product	Amino acid change within the corresponding protein (if exists)	Mutation type	Number of samples seen (among 62 samples)	Percentage among 62 samples
C3037T	ORF1a	Nsp3	106 (F)	Silent	38	61%
C14408T	ORF1b	RNA-dependent RNA polymerase	P323L	Missense	38	61%
A23403G	S	Spike glycoprotein	D614G	Missense	38	61%
G25563T	ORF3a	ORF3a protein	Q57H	Missense	25	40%
G11083T	ORF1a	Nsp6	L37F	Silent	24	38%
C18877T	ORF1b	3’ to 5’ exonuclease	280 (L)	Silent	22	35%
G29742T	3’ UTR				22	35%
G1397A	ORF1a	Nsp2	V198I	Missense	21	33%
T28688C	N	Nucleocapsid phosphoprotein	139(L)	Silent	21	33%
C241T	5’ UTR				20	32%
C26735T	M	Membrane glycoprotein	71(Y)	Silent	13	20%
C26549T	M	Membrane glycoprotein	9(T)	Silent	12	19%
C884T	ORF1a	Nsp2	R27C	Missense	11	17%
G8653T	ORF1a	Nsp4	M33I	Missense	11	17%
G28881A	N	Nucleocapsid phosphoprotein	R203K	Missense	9	14%
G28882A						
G28883C	N	Nucleocapsid phosphoprotein	G204R	Missense	9	14%
C228T	5’ UTR				8	13%
A9514G	ORF1a	Nsp4	320(L)	Silent	7	11%
C22444T	S	Spike glycoprotein	294(D)	Silent	7	11%
G26720C	M	Membrane glycoprotein	66(V)	Silent	7	11%
C28854T	N	Nucleocapsid phosphoprotein	S194L	Missense	7	11%
C5736T	ORF1a	Nsp3	A1006V	Missense	6	10%
G9479T	ORF1a	Nsp4	G309C	Missense	6	10%
T28835C	N	Nucleocapsid phosphoprotein	S188P	Missense	6	10%
C7765T	ORF1a	Nsp3	1682(S)	Silent	5	8%
C17690T	ORF1b	Helicase	S485L	Missense	5	8%
T26551C	M	Membrane glycoprotein	V10A	Missense	5	8%

### 2.2. Nucleotide substitution analysis

SARS-CoV-2 isolate sequences from Turkey were compared to the reference SARS-CoV-2 sequence (NC_045512.1), by means of nucleotide substitutions. The constructed dataset was MAFFT^5^5MAFFT (2020). Multiple alignment program for amino acid or nucleotide sequences [online]. Website https://mafft.cbrc.jp/alignment/software/ [accessed 25 April 2020]. aligned and manually edited using the AliView program to verify that the sequences were in frame. The nucleotide numbers were indicated starting from the 5’ UTR of the viral sequence, while amino acid positions were indicated separately for each corresponding protein coding region4. The positions of the nucleotides and amino acids were further confirmed from GeneBank reference sequences (NC_045512.1)4. The nonconserved nucleotide positions were determined and the nucleotide substitutions were evaluated for their effects on amino acid changes by using the AliView and MEGA software.

## 3. Results

**Figure 1:**
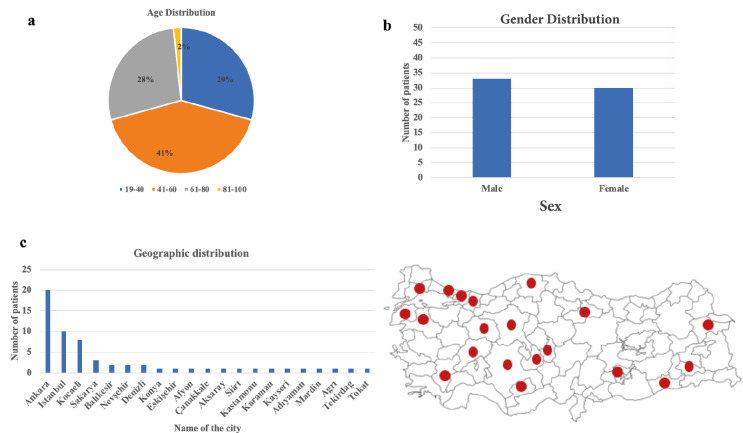
a)The age, b) Sex and c)Geographic distribution of the 62 patients from whom the viral isolates were taken.

The age interval of the patients from Turkey, from whom the viral isolates were taken, was between 19 and 82. 41% of the patients were between the age range of 41–60, while 29% was between 19 and 40 and 28% was between 61 and 80 (Figure 1a) and the sex distribution was approximately equal (Figure 1b). Most of the uploaded samples seem to come from middle-west side of the country (Figure 1c). 

When the SARS-CoV-2 strains from Turkey, compared to the reference viral sequence (NC 045512.1), some missense and silent mutations were identified. In more than 40% of the viral isolates, one or more of nucleotide substitutions of C3037T, C14408T ,A23403G, and G25563T were observed, which are present in the coding regions for the Nsp3, RNA-dependent RNA polymerase, spike glycoprotein and ORF3a protein, respectively (Table 1). Among these mostly seen substitutions, the ones seen in Nsp3, RNA-dependent RNA polymerase and spike glycoprotein were present in 61% (38/62) of the isolates from Turkey. Other viral genome regions where the nucleotide substitutions were observed in 5 or more samples out of 62 include, Nsp6 (24/62), 3’ to 5’ exonuclease (22/62), Nsp2 (22/62), nucleocapsid protein (21/62), membrane glycoprotein (13/62), Nsp4 (11/62), Nsp3 (6/62) and Helicase (5/62) (Table 1).

Each of the 62 viral isolates were also evaluated by means of the mutations they harbor and 24 viral isolates found to have unique mutations in addition to the common mutations they harbor, that are not seen in other isolates from Turkey (Table 2; Supplementary Table 2).

**Table 2:** Nucleotide substitutions present only in a single isolate among the analyzed viral isolates from Turkey.

GISAID ID	Nucleotide substitutions present only in the corresponding isolate
EPI_ISL_424366	G23876A, C29563T
EPI_ISL_427391	C2997T
EPI_ISL_428368	C12809T
EPI_ISL_428717	C21304A, G21305A, C28054T
EPI_ISL_428718	C8782T, G14122T, G28878A
EPI_ISL_428720	G12248T, T23559A
EPI_ISL_428713	C4524T
EPI_ISL429870	C19170T, C25275T
EPI_ISL_429873	C1437T
EPI_ISL_429864	G944A
EPI_ISL429865	C7834T, C26340T
EPI_ISL_429868	C11074T
EPI_ISL_437306	C8683A
EPI_ISL_437307	T6202A, C8964T, C10202T, C16247T, C24865T
EPI_ISL_437308	C15240T
EPI_ISL_437309	C16616T, A23734T
EPI_ISL_437317	G22468T, G25314T, T28144C
EPI_ISL_437318	C5477T, C6402T
EPI_ISL_437319	G19285A
EPI_ISL_437328	C1825T
EPI_ISL_437330	C5826A
EPI_ISL_437331	C12700T
EPI_ISL_437333	T15102C
EPI_ISL_437335	A27354G

Most of the viral isolates found to have different nucleotide substitution combinations. However, the same nucleotide substitutions were observed for the samples EPI_ISL42874 and EPI_ISL_429871. Simlarly, EPI_ISL437411 and EPI_ISL437413 were also found to have the same nucleotide substitution combination among them (Supplementary Table 2). The analyzed viral isolates were found to harbor 4 to12 mutations per isolate compared to the reference sequence (Supplementary Table 2). C > T mutations also observed to predominate among the analyzed viral isolates.

## 4. Discussion

The first COVID-19 case in Turkey was declared in March 11th, almost after two and a half months from the first case declaration in China. When the nucleotide substitutions for the SARS-CoV-2 isolates in Turkey were analyzed compared to the reference genome of the virus from China, it was seen that during this time period, the virus had undergone several nucleotide substitutions, including silent and missense mutations.

When we consider the mutations in ORF1ab, nucleotide substitutions of C884T, G1397A, C3037T, G8653T, G11083T, C14408T, and C18877T were seen in more than 15% (11/62) of the samples. C14408T mutation within the RNA-dependent RNA polymerase encoding region of ORF1b, which is a missense mutation that leads to an amino acid change from proline to leucine at position 323 (P323L) in RNA polymerase protein, was amongst the most commonly seenmutations [61% (38/62)] in isolates from Turkey. Both amino acids seem to have similar isoelectric points and this mutation is mostly seen in isolates from Europe, followed by North America (Pachetti et al., 2020). The mutation was found to be present in European isolates after February 20th, 2020 and thought to be associated with increased number of point mutations compared to isolates from Asia, which proposed to be somehow due to the presence of RNA polymerase within the proofreading machinery of the virus (Pachetti et al., 2020). A recent study indicates that SARS-CoV-2 genomes which harbor C14408T mutation, are more likely to have mutations in the membrane (M) and envelope (E) proteins (Eskier et al., 2020). Furthermore, recently revealed structure of the replicating RNA polymerase of SARS-CoV-2 may further help to understanding of the effect of certain mutations within this protein (Hillen et al., 2020).

G11083T, corresponding to the amino acid substitution L37F within Nsp6 protein, was present in 38% of the samples (24/62) and this mutation was previously seen in SARS-CoV-2 sequences analyzed from all over the world (Benvenuto et al., 2020; Wang et al., 2020). In the study of Wang et al. (2020), C8782T substitution, which is also a silent mutation, was present in 28 out of 95 samples, although this mutation was only present in 2 samples (EPI_ISL_428718 and EPI_ISL_437317) in our study, which indicates that this mutation is not as common as in Europe for the viral isolates in Turkey and may be related with isolates from other countries. Both C8782T and G11083T mutations were found to be mostly present in Oceania isolates, where followed by North America and Europe subsequently (Pachetti et al., 2020). G1397A substitution in Nsp2 encoding region of ORF1a, which was present in 33% (21/62) of the isolates, was mainly seen in viral isolates from Oceania, however was also present in minor amounts in isolates from Asia and North America (Pachetti et al., 2020). This substitution leads to an amino acid change from valine to isoleucine at the position 198 (V198I) within Nsp2 protein, where both amino acids have the same isoelectric points. 

A23403G mutation in the spike glycoprotein coding region was also amongst the mostly seen mutations in viral isolates from Turkey (61%). Spike glycoprotein functions to bind target receptor and facilitate membrane fusion and viral entry (Ou et al., 2020). This protein has 2 subunits, S1 and S2, where the former mediates attachment and the later mediates membrane fusion. A23403G substitution was found to be present in isolates from Europe and leads to an amino acid change from aspartate to glycine at position 614 (D614G) within the spike glycoprotein, where these amino acids differ by means of their isoelectric points (Pachetti et al., 2020). Another mutation found within the spike protein encoding region was C22444T, which is a silent mutation and seen in 7 out of 62 isolates (Table 1).

Similar to SARS-CoV, receptor binding domain (RBD) within the spike glycoprotein of SARS-CoV-2 seems to play a major role in viral infection by acting as an interaction point with target receptors on the host cell surface (Raj et al., 2013). In a recent study which performed multivariate generalized linear model (GLM) analysis with outpatient and hospitalized patients in the Sheffield Teaching Hospitals NHS Foundation Trust as the outcome revealed that patients carrying G614 mutation had higher viral loads compared to D614, although D614G status did not significantly affect the hospitalization status (Korber et al., 2020). It also seems that the viral isolates which carry D614G mutation increases in number across the world and this mutation was proposed to have effect on the viral infectivity either due to its presence on the spike protein promoter surface region which might affect hydrogen bonding properties with neighbouring promoter regions or due to be in a site surrounded by antibody-dependent enhancement targets, where antibody binding may lead to a confirmational change that might increase the ACE2 interaction. Both mechanisms were proposed to play role in a more transmissible form of the virus (Korber et al., 2020). On the other hand, another study perfomed on 15,000 SARS-CoV-2 genomes indicated that the recurrent mutations do not increase transmissibility (Dorp et al., 2020). Therefore, the effect of D614G mutation on the transmission of the virus is still a debate. 

Five out of 27 commonly seen mutations in viral isolates from Turkey were within the nucleocapsid phosphoprotein. One interesting finding was the presence of 2 subsequent missense mutations that are seen as a cluster in 9 out of 62 samples (14 %). These mutations were due to nucleotide substitutions in 3 nucleotides in order where 2 of them (G28881A and G28882A) results in arginine to lysine (R203K) substitution and the third one (G28883C) results in glycine to arginine (G204R) in the nucleocapsid phosphoprotein, where both substituted amino acids differ from their original amino acids in means of their isoelectric points (Pachetti et al., 2020) (Table 1). G28881A, along with A23403G substitution in spike glycoprotein, seems to have occurred after February 16th, 2020 in Europe (Pachetti et al., 2020). C28854T substitution in the nucleocapsid protein coding region, which leads to an amino acid change (S194L), was another missense mutation that also was seen in 6/95 samples in a previous study where 95 sequences from different countries were evaluated (Wang et al., 2020). These findings support the presence of the mutation in several strains all over the world including some isolates from Turkey. In addition to S protein, nucleocapsid protein was also proposed to be important in COVID-19 infectivity (Goh et al, 2020). Further studies are needed to clarify if the missense mutations within this region can be important in the infection strategy of the SARS-CoV-2 virus or not. 

G1397A, T28688C and G29742T substitutions were said to belong to a monophyletic group which is defined by the presence of these 3 mutations and were found to present in patients who were traveled to or are residents in Iran (Eden J et al., 2020) as well as in Australian and New Zealand isolates. Twenty-one viral isolates from Turkey harbor those 3 mutations together (EPI_ISL_417413, EPI_ISL_424366, EPI_ISL_428722, EPI_ISL_428713, EPI_ISL_429872, EPI_ISL_429865, EPI_ISL_429868, EPI_ISL_437319, EPI_ISL_437324, EPI_ISL_437325, EPI_ISL_437326, EPI_ISL_437327, EPI_ISL_437332, EPI_ISL_437306, EPI_ISL_437307, EPI_ISL_437312, EPI_ISL_437314, EPI_ISL_437320, EPI_ISL_437321, EPI_ISL_437322, EPI_ISL_437323, EPI_ISL_437334). The travel history to Iran were only mentioned for 6 samples in GISAID (EPI_ISL_437319, EPI_ISL_437324, EPI_ISL_437325, EPI_ISL_437326, EPI_ISL_437327, EPI_ISL_437332), where 4 of them (EPI_ISL_437319, EPI_ISL_437324, EPI_ISL_437325,EPI_ISL_437327) are known to harbor this monophyletic group. However, other 2 samples do not harbor any of these mutations, although they have travel history to Iran. Identification of detailed epidemiological data of these samples can be important to identify if these patients somehow had contact in relation to any of these countries.

A phylogenetic network analysis of 160 SARS-CoV-2 genomes, identified 3 central variants of the virus (named as A, B and C), compared to the bat coronavirus (Forster et al., 2020). These variants differ from each other by amino acid substitutions. Node A has 2 subclusters where there is T or C in nucleotide position 29095. B-type variant have T8782C nonsynonymous and C28144T synonymous (Leu to Ser) substitution in addition to A-type and C-type variant have G26144T synonymous mutation (Gly to Val) in addition to B-type substitutions. A- and C-types are said to present mainly outside of East Asia, where B-type is said to be present mainly in East Asia. The isolates from Turkey analyzed in this study mainly harbor cytosine in nucleotide position 29095. Another study, which analyzed 622 complete SARS-CoV-2 genomes by an unrooted maximum likelihood tree divided the viral genomes into 3 clusters, which was mainly similar to the 3 viral variants identified in 162 SARS-CoV-2 genomes (Forster et al., 2020), and performed linkage analysis between the mutations seen within these clusters (Bai et al., 2020). According to the linkage analysis, C241T, C3037T, C14408T and A23403G in Cluster 3 were in complete linkage and the TTTG haplotype was high in Europe and correlated with the death rate. In the viral isolates analyzed in this study, C3037T, C14408T and A23403G, which were the most common mutations (61%), exist together. In 11 out of 62 samples, C241T was also observed to be present together with C3037T, C14408T and A23403G. The reason of not observing C241T in linkage with other mutations with the same percentage can be due to the absence of the first 265 nucleotides in 25 of the uploaded sequences to GISAID. However, there are also isolates that harbor either C241T and not the other 3 mutations or vice versa. This haplotype was proposed to be related with the high death rates in Europe (Bai et al., 2020). Analyzing the course of the COVID-19 disease in patients from whom the viral isolates were taken can give further information about the relatedness of this haplotype with the death rates in Turkey.

Apart from the common mutations, when we consider mutations seen in a single sample among analyzed isolates, some are not mentioned previously in the literature. C8782T was previously seen in more than 10 isolates in Guangdong province of China (Lu et al., 2020) and proposed to be clade specific in a study performed on 313 SARS-CoV-2 genomes (Li, Li, Cui, and Wu, 2020). G28878A, which is present in the same isolate with C8782T, was observed in isolates from Australia and USA (Li et al., 2020). Some of the observed mutations can be either unique to corresponding isolate or can also be a result of homoplasy or sequencing artefact since in an ongoing study, some sites within the viral genome are suspected to be homoplasic substitutions or sequencing artefacts^6^6Issues with SARS-CoV-2 sequencing data (2020). [online]. Website https://virological.org/t/issues-with-sars-cov-2-sequencing-data/473. [Accessed 5 May 2020]. G11083T is the most common one among such sites across different countries and sequencing technologies, which might be an indicator of this position being either a site for frequent mutation or an artefact. However, 38% of the samples (24/62) analyzed in this study harbor this mutation, although being sequenced by different technologies, which is consistent with this site being a site for frequent mutation. 

Some homoplasic sites were found to bespecific to certain sequencing technologies, such as the nucleotide position 11074. Nucleotide 3037 and 11074 were reported to be either artefacts or hypermutable low-fitness sites. However, 3037 was found to have a linkage with 3 other mutations and mainly observed in Europe. It is also among the mostly seen mutations along with 14408 and 23403 in the viral isolates analyzed. Therefore, it can be 1 of the hypermutable sites within the viral genome. Apart from 11074 and 3037, detailed analysis of the identified unique mutations by means of possible sequencing artefacts and homoplasic sites can reveal more information about them. Therefore, considering the possible sequencing artefacts while analyzing the sequences for substitution can be important (Korber et al., 2020).

Recombination is known to take place in evolution of coronaviruses and some breakpoints for recombination in SARS-CoV-2 was also reported (Korber et al., 2020; Rehman et al., 2020). Therefore, apart from single nucleotide mutations, identification of possible recombinational events can be important in vaccine development strategies.

In our dataset, 2 samples found to harbor quite lots of mutations, which both are sequenced with the same sequencing technology. Therefore, only the common mutations with other isolates in one of them were considered and the other one was excluded totally since the mutations it harbors were quite extreme. The number of extreme mutations might be due to the use of separate sequencing technology in these strains compared to the other isolates that are analyzed in this study. 

The nucleotide substitutions showed that viral isolates from Turkey are genetically close to the ones from Europe, Middle East, North America and Asia. C3037T, C14408T and A23403G substitutions, which are present in Nsp3, RNA-dependent RNA polymerase and spike encoding regions respectively, were found to be the mostly seen mutations in Turkey SARS-CoV-2 isolates. Considering the missense mutations encountered in these isolates, further studies are needed how the identified amino acid changes affect the structure of the related proteins as well as the infectivity and spread of the virus. Also, the silent mutations within SARS-CoV-2 genome can be followed up to determine if any further missense mutations will take place within these regions, which may be helpful to understand the evolutionary strategy of the virus as it continues to evolve during its spread through the world. 

## Acknowledgments/Disclaimers

The authors acknowledge to all the researchers in originating and submitting labs who have shared the SARS-CoV-2 genome data on GISAID ((http://www.gisaid.org/). The extended acknowledgement can be found as a supplementary file. No funding was used to conduct this research.

## Supporting Information

Supplementary Table 1

**Table:** General information taken from GISAID about the SARS-CoV-2 sequences used in this study. (NA: Not available)

GISAID ID	Location	Sex	Age	Collection date	Submission date	Travel history/other	Sequencing technology
EPI_ISL_429873	Kocaeli	Male	71	2020-03-23	2020-04-24	NA	Illumina Miseq assembly: Burrows-Wheeler aligner v.07.17-r1188 1,000x coverage
EPI_ISL_429872	Kocaeli	Female	50	2020-03-25	2020-04-24	NA	Illumina Miseq assembly: Burrows-Wheeler aligner v.07.17-r1188 1,000x coverage
EPI_ISL_429871	Ankara	Male	77	2020-03-23	2020-04-24	Patient travelled to Saudi Arabia	Illumina Miseq assembly: Burrows-Wheeler aligner v.07.17-r1188 1,000x coverage
EPI_ISL_429870	Sakarya	Female	57	2020-03-22	2020-04024	Patient travelled to Saudi Arabia	Illumina Miseq assembly: Burrows-Wheeler aligner v.07.17-r1188 1,000x coverage
EPI_ISL_429869	Konya	Female	59	2020-03-17	2020-04-24	Patient travelled to Saudi Arabia	IlluminaMiseq assembly: Burrows-Wheeler aligner v.07.17-r1188 1,000x coverage
EPI_ISL_429868	Eskişehir	Female	79	2020-03-17	2020-04-24	NA	Illumina Miseq assembly: Burrows-Wheeler aligner v.07.17-r1188 1,000x coverage
EPI_ISL_429867	Balıkesir	Female	72	2020-03-17	2020-04-24	NA	Illumina Miseq assembly: Burrows-Wheeler aligner v.07.17-r1188 1,000x coverage
EPI_ISL_429866	Afyon	Female	52	2020-03-16	2020-04-24	Patient travelled to Saudi Arabia	IlluminaMiseq Assembly: Burrows-Wheeler Aligner v.07.17-r1188 1,000x coverage
EPI_ISL_429865	Çanakkale	Female	72	2020-03-18	2020-04-24	NA	Illumina Miseq assembly: Burrows-Wheeler aligner v.07.17-r1188 1,000x coverage
EPI_ISL_429864	Sakarya	Male	33	2020-03-22	2020-04-24	NA	Illumina Miseq assembly: Burrows-Wheeler aligner v.07.17-r1188 1,000x coverage
EPI_ISL_429863	Sakarya	Female	42	2020-03-22	2020-04-24	NA	Illumina Miseq assembly: Burrows-Wheeler aligner v.07.17-r1188 1,000x coverage
EPI_ISL_429862	Ankara	Male	65	2020-03-22	2020-04-24	Patient travelled to Saudi Arabia	Illumina Miseq assembly: Burrows-Wheeler aligner v.07.17-r1188 1,000x coverage
EPI_ISL_429861	Ankara	Male	48	2020-03-22	2020-04-24	NA	Illumina Miseq assembly: Burrows-Wheeler aligner v.07.17-r1188 1,000x coverage
EPI_ISL_428723	Aksaray	Male	48	2020-03-22	2020-04-21	Patient travelled to Saudi Arabia	Illumina Miseq assembly: Burrows-Wheeler aligner v.07.17-r1188 1,000x coverage
EPI_ISL_428722	Balıkesir	Female	37	2020-03-22	2020-04-21	NA	Illumina Miseq assembly: Burrows-Wheeler aligner v.07.17-r1188 1,000x coverage
EPI_ISL_428721	Ankara	Male	NA	2020-03-21	2020-04-21	NA	Illumina Miseq assembly: Burrows-Wheeler aligner v.07.17-r1188 1,000x coverage
EPI_ISL_428720	Ankara	Female	35	2020-03-21	2020-04-21	NA	Illumina Miseq assembly: Burrows-Wheeler aligner v.07.17-r1188 1,000x coverage
EPI_ISL_428719	Siirt	Male	52	2020-03-21	2020-04-21	NA	Illumina Miseq assembly: Burrows-Wheeler aligner v.07.17-r1188 1,000x coverage
EPI_ISL_428718	Kocaeli	Male	35	2020-03-19	2020-04-21	Patient travelled to Saudi Arabia	Illumina Miseq assembly: Burrows-Wheeler aligner v.07.17-r1188 1,000x coverage
EPI_ISL_428717	Kocaeli	Male	38	2020-03-19	2020-04-21	NA	Illumina Miseq assembly: Burrows-Wheeler aligner v.07.17-r1188 1,000x coverage
EPI_ISL_428716	Ankara	Female	62	2020-03-18	2020-04-21	Patient travelled to Saudi Arabia	Illumina Miseq assembly: Burrows-Wheeler aligner v.07.17-r1188 1,000x coverage
EPI_ISL_428715	Nevşehir	Female	55	2020-03-18	2020-04-21	NA	Illumina Miseq assembly: Burrows-Wheeler aligner v.07.17-r1188 1,000x coverage
EPI_ISL_428714	Kastamonu	Male	60	2020-03-18	2020-04-21	Patient travelled to Saudi Arabia	Illumina Miseq assembly: Burrows-Wheeler aligner v.07.17-r1188 1,000x coverage
EPI_ISL_428713	Ankara	Female	NA	2020-03-18	2020-04-21	NA	Illumina Miseq assembly: Burrows-Wheeler aligner v.07.17-r1188 1,000x coverage
EPI_ISL_428712	Karaman	Male	72	2020-03-17	2020-04-21	Patient travelled to France	Illumina Miseq assembly: Burrows-Wheeler aligner v.07.17-r1188 1,000x coverage
EPI_ISL_428368	İstanbul	Female	49	2020-04-16	2020-04-20	NA	IlluminaNextSeq Assembly: BWA-MEM1,750x coverage
EPI_ISL_428346	İstanbul	Male	49	2020-04-17	2020-04-20	NA	Illumina Next Seq assembly: BWA-MEM 2,350x coverage
EPI_ISL_427391	İstanbul	Male	51	2020-04-13	2020-04-18	NA	Illumina Next Seq assembly: BWA-MEM 0.7.17.15,845x coverage
EPI_ISL_424366	Kayseri	Male	82	2020-03-17	2020-04-13	NA	Illumina Miseq assembly: Burrows-Wheeler aligner v.07.17-r1188 26,200x coverage
EPI_ISL_417413	NA	Female	27	2020-03-17	2020-03-25	NA	Nanopore MinIONGeneious Prime245X coverage
EPI_ISL_435057	Adıyaman	Male	80	2020-04-09	2020-05-02	NA	Oxford Nanopore MinION assembly: Geneious Prime 2020.1.240X coverage
EPI_ISL_437304	Nevşehir	Female	54	2020-03-26	2020-05-08	NA	Oxford Nanopore GridION assembly: Geneious Prime1000X coverage
EPI_ISL_437305	Kocaeli	Female	66	2020-03-27	2020-05-08	NA	Oxford Nanopore GridION assembly: Geneious Prime1000X coverage
EPI_ISL_437306	Kocaeli	Male	30	2020-03-27	2020-05-08	NA	Oxford Nanopore GridION assembly: Geneious Prime1000X coverage
EPI_ISL_437307	Mardin	Female	19	2020-03-25	2020-05-08	NA	Oxford Nanopore GridION assembly: Geneious Prime1000X coverage
EPI_ISL_437308	Ankara	Male	54	2020-03-25	2020-05-08	NA	Oxford Nanopore GridION assembly: Geneious Prime1000X coverage
EPI_ISL_437309	Ankara	Female	58	2020-03-26	2020-05-08	NA	Oxford Nanopore GridION assembly: Geneious Prime1000X coverage
EPI_ISL_437310	Ankara	Male	NA	2020-03-27	2020-05-08	NA	Oxford Nanopore GridION assembly: Geneious Prime1000X coverage
EPI_ISL_437311	Ankara	Male	61	2020-03-27	2020-05-08	NA	Oxford Nanopore GridION assembly: Geneious Prime1000X coverage
EPI_ISL_437312	Kocaeli	Male	52	2020-03-25	2020-05-08	NA	Oxford Nanopore GridION assembly: Geneious Prime1000X coverage
EPI_ISL_437313	Kocaeli	Male	49	2020-03-27	2020-05-08	NA	Oxford Nanopore GridION assembly: Geneious Prime1000X coverage
EPI_ISL_437314	Ankara	Male	62	2020-03-26	2020-05-08	NA	Oxford Nanopore GridION assembly: Geneious Prime1000X coverage
EPI_ISL_437315	Ankara	Female	33	2020-03-26	2020-05-08	NA	Oxford Nanopore GridION assembly: Geneious Prime1000X coverage
EPI_ISL_437316	Denizli	Female	NA	2020-03-25	2020-05-08	NA	Oxford Nanopore GridION assembly: Geneious Prime1000X coverage
EPI_ISL_437317	Ankara	Female	58	2020-03-27	2020-05-08	Patient travelled to Saudi Arabia	Oxford Nanopore GridION assembly: Geneious Prime1000X coverage
EPI_ISL_437318	Ankara	Male	31	2020-03-19	2020-05-08	NA	Oxford Nanopore GridION assembly: Geneious Prime1000X coverage
EPI_ISL_437319	Kocaeli	Male	47	2020-03-19	2020-05-08	Patient travelled to Iran	Oxford Nanopore GridION assembly: Geneious Prime1000X coverage
EPI_ISL_437320	İstanbul	Female	41	2020-03-19	2020-05-08	NA	Oxford Nanopore GridION assembly: Geneious Prime1000X coverage
EPI_ISL_437321	İstanbul	Female	25	2020-03-19	2020-05-08	NA	Oxford Nanopore GridION assembly: Geneious Prime1000X coverage
EPI_ISL_437322	Ankara	Female	62	2020-03-19	2020-05-08	NA	Oxford Nanopore GridION assembly: Geneious Prime1000X coverage
EPI_ISL_437323	İstanbul	Male	41	2020-03-19	2020-05-08	Patient travelled toTaiwan	Oxford Nanopore GridION assembly: Geneious Prime1000X coverage
EPI_ISL_437324	İstanbul	Male	27	2020-03-19	2020-05-08	Patient travelled to Iran	Oxford Nanopore GridION assembly: Geneious Prime1000X coverage
EPI_ISL_437325	İstanbul	Male	44	2020-03-19	2020-05-08	Patient travelled to Iran	Oxford Nanopore GridION assembly: Geneious Prime1000X coverage
EPI_ISL_437326	İstanbul	Male	38	2020-03-19	2020-05-08	Patient travelled to Iran	Oxford Nanopore GridION assembly: Geneious Prime1000X coverage
EPI_ISL_437327	Ağrı	Female	35	2020-03-19	2020-05-08	Patient travelled to Iran	Oxford Nanopore GridION assembly: Geneious Prime1000X coverage
EPI_ISL_437328	Tekirdağ	Female	35	2020-03-19	2020-05-08	Patient travelled to Saudi Arabia	Oxford Nanopore GridION assembly: Geneious Prime1000X coverage
EPI_ISL_437329	Ankara	Male	31	2020-03-19	2020-05-08	Patient travelled to Saudi Arabia	Oxford Nanopore GridION assembly: Geneious Prime1000X coverage
EPI_ISL_437330	Tokat	Male	20	2020-03-19	2020-05-08	Health worker	Oxford Nanopore GridION assembly: Geneious Prime1000X coverage
EPI_ISL_437331	Ankara	Female	59	2020-03-25	2020-05-08	Patient travelled to Saudi Arabia	Oxford Nanopore GridION assembly: Geneious Prime1000X coverage
EPI_ISL_437332	İstanbul	Male	50	2020-03-18	2020-05-08	Patient travelled to Iran	Oxford Nanopore GridION assembly: Geneious Prime1000X coverage
EPI_ISL_437333	Ankara	Male	64	2020-03-25	2020-05-08	NA	Oxford Nanopore GridION assembly: Geneious Prime1000X coverage
EPI_ISL_437334	Ankara	Female	64	2020-03-24	2020-05-08	NA	Oxford Nanopore GridION assembly: Geneious Prime1000X coverage
EPI_ISL_437335	Denizli	Female	79	2020-03-25	2020-05-08	NA	Oxford Nanopore GridION assembly: Geneious Prime1000X coverage

Supplementary Table 2

**Table:** Supplementary Table2. Nucleotide substitutions present in 62 SARS-CoV-2 viral genomes fromTurkey (submitted to GISAID between March 25th and May 22nd 2020) compared to the SARS-CoV-2 reference genome NC 045512.1. The nucleotide positions are given starting from 5’UTR. The star mark (*) indicates mutations that are only seen in the corresponding viral isolate.

GISAID_sample accession ID	Nucleotide substitution at the given position	Corresponding viral gene	Corresponding viral gene product
			
EPI_ISL_417413	*T580A	ORF1ab	Leader protein
	*G779C		
	*T946A	ORF1ab	Nsp2
	*T1100G		
	*C1101T		
	*A1106T		
	*A1119C		
	*A1134T		
	*G1156A		
	*G1210A		
	*C1225A		
	*T1359C		
	G1397A		
	*C1420T		
	*G1470A		
	*C1473T		
	*A1475C		
	*G2250A		
	C2455T		
	*A2475T		
	*G2549C		
	*T2586A		
	*G2591A		
	*G2612C		
	*G2715T		
	*A2932G	ORF1ab	Nsp3
	C3117T		
	*G3146 C		
	C3787T		
	C4084T		
	*C7392T		
	*C11232T	ORF1ab	Nsp6
	*G11234A		
	*C13476T	ORF1ab	RNA-dependent RNA polymerase
	*C13492T		
	*C14286T		
	*G14310A		
	*T14394A		
	*C14407A		
	*G14430A		
	*G14443T		
	*T14682G		
	*G14710A		
	*T14740C		
	*C14763A		
	*G14773T		
	*T14808A		
	*C15101A		
	*T15119A		
	*G15958A		
	*C19763A	ORF1ab	EndoRNAse
	*T26396A	E	Envelope
	*T26551C	M	Membrane glycoprotein
	*C26753T		
	C27103T		
	G28109T	ORF8	ORF8 protein
	T28688C	N	Nucleocapsid phosphoprotein
	G29742T		3’UTR stem loop II like motif
			
EPI_ISL_424366	G1397A	ORF1ab	Nsp2
	G11083T	ORF1ab	Nsp6
	*G23876A	S	Spike glycoprotein
	T28688C	N	Nucleocapsid phosphoprotein
	*C29563T		
	G29742T		3’UTR stem loop II like motif
			
EPI_ISL_427391	C2113T	ORF1ab	Nsp2
	*C2997T	ORF1ab	Nsp3
	C3037T		
	C7765T		
	C14408T	ORF1ab	RNA-dependent RNA polymerase
	C17690T	ORF1ab	Helicase
	C18877T	ORF1ab	3’ to 5’ exonuclease
	A23403G	S	Spike glycoprotein
	G25563T	ORF3a	ORF3a protein
			
EPI_ISL_428368	C3037T	ORF1ab	Nsp3
	G11083T	ORF1ab	Nsp6
	*C12809T	ORF1ab	Nsp9
	C14408T	ORF1ab	RNA-dependent RNA polymerase
	A23403G	S	Spike glycoprotein
	G28881A	N	Nucleocapsid phosphoprotein
	G28882A		
	G28883C		
			
EPI_ISL_428346	C2113T	ORF1ab	Nsp2
	C3037T	ORF1ab	Nsp3
	C7765T		
	C14408T	ORF1ab	RNA-dependent RNA polymerase
	C17690T	ORF1ab	Helicase
	C18877T	ORF1ab	3’ to 5’exonuclease
	*G21452T	ORF1ab	2’-O-Ribose methyltransferase
	A23403G	S	Spike protein
	G25563T	ORF3a	ORF3a protein
			
EPI_ISL_428717	C3037T	ORF1ab	Nsp3
	C12741T	ORF1ab	Nsp8
	C14408T	ORF1ab	RNA-dependent RNA polymerase
	C18877T	ORF1ab	3’ to 5’ exonuclease
	*C21304A	ORF1ab	2’-O-Ribose methyltransferase
	*G21305A		
	A23403G	S	Spike protein
	G25563T	ORF3a	ORF3a protein
	C26735T	M	Membrane glycoprotein
	*C28054T	ORF8	ORF8 protein
			
EPI_ISL_428716	C3037T	ORF1ab	Nsp3
	C14408T	ORF1ab	RNA-dependent RNA polymerase
	C18877T	ORF1ab	3’ to 5’ exonuclease
	C22444T	S	Spike glycoprotein
	A23403G		
	G25563T	ORF3a	ORF3a protein
	C26735T	M	Membrane glycoprotein
	C28854T	N	Nucleocapsid phosphoprotein
			
EPI_ISL_428719	C3037T	ORF1ab	Nsp3
	C7765T		
	C14408T	ORF1ab	RNA-dependent RNA polymerase
	C17690T		
	C18877T	ORF1ab	3’ to 5’ exonuclease
	A23403G	S	Spike glycoprotein
	G25563T	ORF3a	ORF3a protein
			
EPI_ISL_428718	C8782T	ORF1ab	Nsp4
	*G14122T	ORF1ab	RNA-dependent RNA polymerase
	G28878A	N	Nucleocapsid phosphoprotein
	G29742T		3’UTR stem loop II like motif
			
EPI_ISL_428720	C3037T	ORF1ab	Nsp3
	*G12248T	ORF1ab	Nsp8
	C14408T	ORF1ab	RNA-dependent RNA polymerase
	C18877T	ORF1ab	3’ to 5’ exonuclease
	A23403G	S	Spike glycoprotein
	*T23559A		
	G25563T	ORF3a	ORF3a protein
	C26735T	M	Membrane glycoprotein
			
EPI_ISL_428722	C884T	ORF1ab	Nsp2
	G1397A		
	G8653T	ORF1ab	Nsp4
	G11083T	ORF1ab	Nsp6
	C12741T	ORF1ab	Nsp9
	T28688C	N	Nucleocapsid phosphoprotein
	G29742T		3’UTR stem loop II like motif
			
EPI_ISL_428721	C3037T	ORF1ab	Nsp3
	C14178T	ORF1ab	RNA-dependent RNA polymerase
	C14408T		
	C18877T	ORF1ab	3’ to 5’ exonuclease
	A23403G	S	Spike glycoprotein
	G25563T	ORF3a	ORF3a protein
	G26718T	M	Membrane glycoprotein
	C26735T		
			
EPI_ISL_428723	G881A	ORF1ab	Nsp2
	C3037T	ORF1ab	Nsp3
	C14408T	ORF1ab	RNA-dependent RNA polymerase
	C18877T	ORF1ab	3’ to 5’ exonuclease
	C22444T	S	Spike glycoprotein
	A23403G		
	G25563T	ORF3a	ORF3a protein
	C26735T	M	Membrane glycoprotein
	C28854T	N	Nucleocapsid phosphoprotein
			
EPI_ISL_428713	G1397A	ORF1ab	Nsp2
	C2455T		
	C3117T	ORF1ab	Nsp3
	C3787T		
	C4084T		
	*C4524T		
	G11083T	ORF1ab	Nsp6
	G28109T	ORF8	ORF8 protein
	T28688C	N	Nucleocapsid phosphoprotein
	G29742T		3’UTR stem loop II like motif
			
EPI_ISL_428712	C2416T	ORF1ab	Nsp2
	C3037T	ORF1ab	Nsp3
	G8371T		
	G11083T	ORF1ab	Nsp6
	C14408T	ORF1ab	RNA-dependent RNA polymerase
	A23403G	S	Spike glycoprotein
	G25563T	ORF3a	ORF3a protein
			
EPI_ISL_428715	C3037T	ORF1ab	Nsp3
	C14178T	ORF1ab	RNA-dependent RNA polymerase
	C14408T		
	C18877T	ORF1ab	3’ to 5’ exonuclease
	A23403G	S	Spike glycoprotein
	G25563T	ORF3a	ORF3a protein
	G26718T	M	Membrane glycoprotein
	C26735T		
			
EPI_ISL_428714	C3037T	ORF1ab	Nsp3
	C14408T	ORF1ab	RNA-dependent RNA polymerase
	C18877T	ORF1ab	3’ to 5’ exonuclease
	A23403G	S	Spike glycoprotein
	G25563T	ORF3a	ORF3a protein
	C26735T	M	Membrane glycoprotein
			
EPI_ISL_429871	C3037T	ORF1ab	Nsp3
	C14408T	ORF1ab	RNA-dependent RNA polymerase
	C18877T	ORF1ab	3’ to 5’ exonuclease
	A23403G	S	Spike glycoprotein
	G25563T	ORF3a	ORF3a protein
	C26735T	M	Membrane glycoprotein
			
EPI_ISL_429870	C3037T	ORF1ab	Nsp3
	C14408T	ORF1ab	RNA-dependent RNA polymerase
	*C19170T	ORF1ab	3’ to 5’ exonuclease
	A23403G	S	Spike glycoprotein
	*C25275T		
	G28881A	N	Nucleocapsid phosphoprotein
	G28882A		
	G28883C		
			
EPI_ISL_429873	*C1437T	ORF1ab	Nsp2
	C3037T	ORF1ab	Nsp3
	C14408T	ORF1ab	RNA-dependent RNA polymerase
	A23403G	S	Spike glycoprotein
	G28881A	N	Nucleocapsid phosphoprotein
	G28882A		
	G28883C		
			
EPI_ISL_429872	C884T	ORF1ab	Nsp2
	G1397A		
	G8653T	ORF1ab	Nsp4
	G11083T	ORF1ab	Nsp6
	T28688C	N	Nucleocapsid phosphoprotein
	G29742T		3’UTR stem loop II like motif
			
EPI_ISL_429862	C3037T	ORF1ab	Nsp3
	C14408T	ORF1ab	RNA-dependent RNA polymerase
	A20268G	ORF1ab	endoRNAse
	A23403G	S	Spike glycoprotein
			
EPI_ISL_429861	C3037T	ORF1ab	Nsp3
	C14408T	ORF1ab	RNA-dependent RNA polymerase
	C18877T	ORF1ab	3’ to 5’ exonuclease
	C22444T	S	Spike glycoprotein
	A23403G		
	G25563T	ORF3a	ORF3a protein
	C26735T	M	Membrane glycoprotein
	C28854T	N	Nucleocapsid phosphoprotein
			
EPI_ISL_429864	*G944A	ORF1ab	Nsp2
	C3037T	ORF1ab	Nsp3
	C14408T	ORF1ab	RNA-dependent RNA polymerase
	A23403G	S	Spike glycoprotein
	G28881A	N	Nucleocapsid phosphoprotein
	G28882A		
	G28883C		
			
EPI_ISL_429863	G881A	ORF1ab	Nsp2
	C3037T	ORF1ab	Nsp3
	C14408T	ORF1ab	RNA-dependent RNA polymerase
	C18877T	ORF1ab	3’ to 5’ exonuclease
	C22444T	S	Spike glycoprotein
	A23403G		
	G25563T	ORF3a	ORF3a protein
	C26735T	M	Membrane glycoprotein
	C28854T	N	Nucleocapsid phosphoprotein
			
EPI_ISL_429866	C3037T	ORF1ab	Nsp3
	C14408T	ORF1ab	RNA-dependent RNA polymerase
	C18877T	ORF1ab	3’ to 5’ exonuclease
	A23403G	S	Spike glycoprotein
	G25563T	ORF3a	ORF3a protein
	C26735T	M	Membrane glycoprotein
			
EPI_ISL_429865	G1397A	ORF1ab	Nsp2
	*C7834T	ORF1ab	Nsp3
	G8653T	ORF1ab	Nsp4
	G11083T	ORF1ab	Nsp6
	*C26340T	E	Envelope
	T28688C	N	Nucleocapsid phosphoprotein
	G29742T		3’UTR stem loop II like motif
			
EPI_ISL_429868	C884T	ORF1ab	Nsp2
	G1397A		
	G8653T	ORF1ab	Nsp4
	C10702T	ORF1ab	3C-like proteinase
	*C11074T	ORF1ab	Nsp6
	G11083T		
	T28688C	N	Nucleocapsid phosphoprotein
	G29742T		3’UTR stem loop II like motif
			
EPI_ISL_429867	C884T	ORF1ab	Nsp2
	C3037T	ORF1ab	Nsp3
	C14408T	ORF1ab	RNA-dependent RNA polymerase
	C18877T	ORF1ab	3’ to 5’ exonuclease
	A23403G	S	Spike glycoprotein
	G25563T	ORF3a	ORF3a protein
	C26735T	M	Membrane glycoprotein
			
EPI_ISL_429869	C3037T	ORF1ab	Nsp3
	C14408T	ORF1ab	RNA-dependent RNA polymerase
	C18877T	ORF1ab	3’ to 5’ exonuclease
	C22444T	S	Spike glycoprotein
	A23403G		
	G25563T	ORF3a	ORF3a protein
	C26735T	M	Membrane glycoprotein
	C28854T	N	Nucleocapsid phosphoprotein
			
EPI_ISL_437304	C241T	5’ UTR	
	C2113T	ORF1ab	Nsp2
	C3037T	ORF1ab	Nsp3
	C7765T		
	C14408T	ORF1ab	RNA-dependent RNA polymerase
	C17690T	ORF1ab	Helicase
	C18877T	ORF1ab	3’ to 5’ exonuclease
	A23403G	S	Spike glycoprotein
	*C25549T	ORF3a	ORF3a protein
	G25563T		
			
EPI_ISL_437305	C241T	5’UTR	
	C3037T	ORF1ab	Nsp3
	C14408T	ORF1ab	RNA-dependent RNA polymerase
	A23403G	S	Spike glycoprotein
	C26549T	M	Membrane glycoprotein
	G28881A	N	Nucleocapsid phosphoprotein
	G28882A		
	G28883C		
			
EPI_ISL_437306	G1397A	ORF1ab	Nsp2
	G8653T	ORF1ab	Nsp4
	*C8683A		
	G11083T	ORF1ab	Nsp6
	A23403G	S	Spike glycoprotein
	T28688C	N	Nucleocapsid phosphoprotein
	G29742T		3’UTR stem loop II like motif
		ORF1ab	Nsp2
EPI_ISL_437307	*C1314T	ORF1ab	Nsp2
	G1397A		
	*T6202A	ORF1ab	Nsp2
	*C8964T	ORF1ab	Nsp4
	*C10202T	ORF1ab	3C-like proteinase
	1G1083T	ORF1ab	Nsp6
	C13481T	ORF1ab	Nsp10
	*C16247T	ORF1ab	Helicase
	*C24865T	S	Spike glycoprotein
	T28688C	N	Nucleocapsid phosphoprotein
	G29742T		3’UTR stem loop II like motif
			
EPI_ISL_437308	C241T	5’ UTR	
	C3037T	ORF1ab	Nsp3
	C14408T	ORF1ab	RNA-dependent RNA polymerase
	*C15240T		
	T19839C	ORF1ab	EndoRNase
	A23403G	S	Spike glycoprotein
	C26256T	E	Envelope
	G28881A	N	Nucleocapsid phosphoprotein
	G28882A		
	G28883C		
			
EPI_ISL_437309	C241T	5’ UTR	
	C1059T	ORF1ab	Nsp2
	C3037T	ORF1ab	Nsp3
	C3903T		
	C14408T	ORF1ab	RNA-dependent RNA polymerase
	*C16616T	ORF1ab	Helicase
	A23403G	S	Spike glycoprotein
	*A23734T		
	G25563T	ORF3a	ORF3a protein
			
EPI_ISL_437310	C241T	5’ UTR	
	C3037T	ORF1ab	Nsp3
	C14408T	ORF1ab	RNA-dependent RNA polymerase
	C18877T	ORF1ab	3’ to 5’ exonuclease
	C22444T	S	Spike glycoprotein
	A23403G		
	G25563T	ORF3a	ORF3a protein
	C28854T	N	Nucleocapsid phosphoprotein
			
EPI_ISL_437311	C241T	5’ UTR	
	C3037T	ORF1ab	Nsp3
	C14408T	ORF1ab	RNA-dependent RNA polymerase
	A23403G	S	Spike glycoprotein
	G28881A	N	Nucleocapsid phosphoprotein
	G28882A		
	G28883C		
			
EPI_ISL_437312	C1397T	ORF1ab	Nsp2
	T5182C	ORF1ab	Nsp3
	G8653T	ORF1ab	Nsp4
	G11083T	ORF1ab	Nsp6
	T28688C	N	Nucleocapsid phosphoprotein
	G29742T		3’UTR stem loop II like motif
			
EPI_ISL_437313	C241T	5’ UTR	
	C3037T	ORF1ab	Nsp3
	C14408T	ORF1ab	RNA dependent RNA polymerase
	A23403G	S	Spike glycoprotein
	G28881A	N	Nucleocapsid phosphoprotein
	G28882A		
	G28883C		
			
EPI_ISL_437314	G1397A	ORF1ab	Nsp2
	T5182C	ORF1ab	Nsp3
	C5736T		
	G8653T	ORF1ab	Nsp4
	G11083T	ORF1ab	Nsp6
	C23874T	S	Spike glycoprotein
	T28688C	N	Nucleocapsid phosphoprotein
	G29742T		3’UTR stem loop II like motif
			
EPI_ISL_437315	C1059T	ORF1ab	Nsp2
	C3037T	ORF1ab	Nsp3
	G11083T	ORF1ab	Nsp6
	C14408T	ORF1ab	RNA-dependent RNA polymerase
	A23403G	S	Spike glycoprotein
	G25563T	ORF3a	ORF3a protein
			
EPI_ISL_437316	C241T	5’ UTR	
	C3037T	ORF1ab	Nsp3
	C5736 T		
	C14408T	ORF1ab	RNA-dependent RNA polymerase
	A20268G	ORF1ab	EndoRNase
	A23403G	S	Spike glycoprotein
	C23874T		
			
EPI_ISL_437317	C24T	5’ UTR	
	C8782T	ORF1ab	Nsp4
	*G22468T	S	Spike glycoprotein
	*G25314T		
	*T28144C	ORF8	ORF8 protein
	G28878A	N	Nucleocapsid phosphoprotein
			
EPI_ISL_437318	G1397A	ORF1ab	Nsp2
	T5182C	ORF1ab	Nsp3
	*C5477T		
	C5736T		
	*C6402T		
	G8653T	ORF1ab	Nsp4
	G11083T	ORF1ab	Nsp6
	T28688C	N	Nucleocapsid phosphoprotein
	G29742T		3’UTR stem loop II like motif
			
EPI_ISL_437319	C228T	5’ UTR	
	G1397A	ORF1ab	Nsp2
	G9479T	ORF1ab	Nsp4
	A9514G		
	G11083T	ORF1ab	Nsp6
	*G19285A	ORF1ab	3’ to 5’ exonuclease
	C21789T	S	Spike glycoprotein
	C26549T	M	Membrane glycoprotein
	G26720C		
	T28688C	N	Nucleocapsid phosphoprotein
	T28835C		
	G29742T		3’UTR stem loop II like motif
			
EPI_ISL_437320	C228T	5’ UTR	
	G1397A	ORF1ab	Nsp2
	G9479T	ORF1ab	Nsp4
	A9514G		
	G11083T	ORF1ab	Nsp6
	C26549T	M	Membrane glycoprotein
	G26720C		
	T28688C	N	Nucleocapsid phosphoprotein
	T28835C		
	G29742T		3’UTR stem loop II like motif
			
EPI_ISL_437321	C228T	5’ UTR	
	G1397A	ORF1ab	Nsp2
	G9479T	ORF1ab	Nsp4
	A9514G		
	G11083T	ORF1ab	Nsp6
	C26549T	M	Membrane glycoprotein
	G26720C		
	T28688C	N	Nucleocapsid phosphoprotein
	T28835C		
	G29742T		3’UTR stem loop II like motif
			
EPI_ISL_437322	C241T	5’ UTR	
	G1397A	ORF1ab	Nsp2
	G8653T	ORF1ab	Nsp4
	C10702T	ORF1ab	3C-like proteinase
	G11083T	ORF1ab	Nsp6
	A22964G	S	Spike glycoprotein
	C26549T	M	Membrane glycoprotein
	T26551C		
	T28688C	N	Nucleocapsid phosphoprotein
	G29742T		3’UTR stem loop II like motif
			
EPI_ISL_437323	C241T	5’ UTR	
	G1397A	ORF1ab	Nsp2
	G8653T	ORF1ab	Nsp4
	C10702T	ORF1ab	3C-like proteinase
	G11083T	ORF1ab	Nsp6
	A22964G	S	Spike glycoprotein
	C26549T	M	Membrane glycoprotein
	T28688C	N	Nucleocapsid phosphoprotein
	G29742T		3’UTR stem loop II like motif
			
EPI_ISL_437324	G1397A	ORF1ab	Nsp2
	G8653T	ORF1ab	Nsp4
	C10702T	ORF1ab	3C-like proteinase
	G11083T	ORF1ab	Nsp6
	A22964G	S	Spike glycoprotein
	C26549T	M	Membrane glycoprotein
	T26551C		
	T28688C	N	Nucleocapsid phosphoprotein
	G29742T		3’UTR stem loop II like motif
			
EPI_ISL_437325	C241T	5’ UTR	
	G1397A	ORF1ab	Nsp2
	C5736T	ORF1ab	Nsp3
	G9479T	ORF1ab	Nsp4
	A9514G		
	G11083T	ORF1ab	Nsp6
	C21789T	S	Spike glycoprotein
	G26720C	M	Membrane glycoprotein
	T28688C	N	Nucleocapsid phosphoprotein
	T28835C		
	G29742T		3’UTR stem loop II like motif
			
EPI_ISL_437326	C241T	5’ UTR	
	C3037T	ORF1ab	Nsp3
	C5736T	ORF1ab	Nsp3
	C14408T	ORF1ab	RNA-dependent RNA polymerase
	C18877T	ORF1ab	3’ to 5’ exonuclease
	C22444T	S	Spike glycoprotein
	G25563T	ORF3a	ORF3a protein
	C26256T	E	Envelope
	C28854T	N	Nucleocapsid phosphoprotein
			
EPI_ISL_437327	C241T	5’ UTR	
	G1397A	ORF1ab	Nsp2
	G9479T	ORF1ab	Nsp4
	A9514G		
	G11083T	ORF1ab	Nsp6
	G26720C	M	Membrane glycoprotein
	T28688C	N	Nucleocapsid phosphoprotein
	T28835C		
	G29742T		3’UTR stem loop II like motif
			
EPI_ISL_437328	C241T	5’ UTR	
	*C1825T	ORF1ab	Nsp2
	C2113T		
	C3037T	ORF1ab	Nsp3
	C7765T		
	C14408T	ORF1ab	RNA-dependent RNA polymerase
	C17690T	ORF1ab	Helicase
	C18877T	ORF1ab	3’to 5’ exonuclease
	A23403G	S	Spike glycoprotein
	G25563T	ORF3a	ORF3a protein
			
EPI_ISL_437329	C228T	5’ UTR	
	C5736T	ORF1ab	Nsp3
	G9479T	ORF1ab	Nsp4
	A9514G		
	C13481T	ORF1ab	RNA-dependent RNA polymerase
	C18877T	ORF1ab	3’ to 5’ exonuclease
	G26720C	M	Membrane glycoprotein
			
EPI_ISL_437330	C241T	5’ UTR	
	C3037T	ORF1ab	Nsp3
	*C5826A	ORF1ab	Nsp3
	C14408T	ORF1ab	RNA dependent RNA polymerase
	C18877T	ORF1ab	3’to 5’ exonuclease
	A23403G	S	Spike glycoprotein
	G25563T	ORF3a	ORF3a protein
	C26549T	M	Membrane glycoprotein
			
EPI_ISL_437331	C228T	5’ UTR	
	C3037T	ORF1ab	Nsp3
	*C12700T	ORF1ab	Nsp9
	C14408T	ORF1ab	RNA-dependent RNA polymerase
	C19484T	ORF1ab	3’to 5’ exonuclease
	A20268G	ORF1ab	EndoRNase
	A23403G	S	Spike glycoprotein
	C23874T		
	C26549T	M	Membrane glycoprotein
	T26551C		
	C29741T		3’UTR stem loop II like motif
			
EPI_ISL_437332	C228T	5’ UTR	
	C2416T	ORF1ab	Nsp2
	C3037T	ORF1ab	Nsp3
	G8371T	ORF1ab	Nsp3
	G11083T	ORF1ab	Nsp6
	C14408T	ORF1ab	RNA-dependent RNA polymerase
	G25563T	ORF3a	ORF3a protein
	C26549T	M	Membrane glycoprotein
	C27103T		
			
EPI_ISL_437333	C3037T	ORF1ab	Nsp3
	C14408T	ORF1ab	RNA-dependent RNA polymerase
	*T15102C		
	T19839C	ORF1ab	EndoRNase
	A23403G	S	Spike glycoprotein
	C26549T	M	Membrane glycoprotein
	G28881A	N	Nucleocapsid phosphoprotein
	G28882A		
	G28883C		
			
EPI_ISL_437334	C228T	5’ UTR	
	G1397A	ORF1ab	Nsp2
	C3903T	ORF1ab	Nsp3
	A9514G	ORF1ab	Nsp4
	G11083T	ORF1ab	Nsp6
	*A13376G	ORF1ab	Nsp10
	C13481T	ORF1ab	RNA-dependent RNA polymerase
	C19484T	ORF1ab	3’to 5’ exonuclease
	G26720C	M	Membrane glycoprotein
	T28688C	N	Nucleocapsid phosphoprotein
	T28835C		
	G29742T		3’UTR stem loop II like motif
			
EPI_ISL_437335	C228T	5’ UTR	
	C3037T	ORF1ab	Nsp3
	C3903T	ORF1ab	Nsp3
	C14408T	ORF1ab	RNA-dependent RNA polymerase
	A23403G	S	Spike glycoprotein
	C26549T	M	Membrane glycoprotein
	T26551C		
	*A27354G	ORF6	ORF6 protein
